# Applications of Vertebrate Models in Studying Prostatitis and Inflammation-Associated Prostatic Diseases

**DOI:** 10.3389/fmolb.2022.898871

**Published:** 2022-07-05

**Authors:** Joosje Bleeker, Zhu A. Wang

**Affiliations:** Department of Molecular, Cell, and Developmental Biology, University of California, Santa Cruz, Santa Cruz, CA, United States

**Keywords:** mouse model, prostatitis, prostate cancer, BPH, chronic inflammation

## Abstract

It has long been postulated that the inflammatory environment favors cell proliferation, and is conducive to diseases such as cancer. In the prostate gland, clinical data implicate important roles of prostatitis in the progression of both benign prostatic hyperplasia (BPH) and prostate cancer (PCa). However, their causal relationships have not been firmly established yet due to unresolved molecular and cellular mechanisms. By accurately mimicking human disease, vertebrate animals provide essential *in vivo* models to address this question. Here, we review the vertebrate prostatitis models that have been developed and discuss how they may reveal possible mechanisms by which prostate inflammation promotes BPH and PCa. Recent studies, particularly those involving genetically engineered mouse models (GEMMs), suggest that such mechanisms are multifaceted, which include epithelium barrier disruption, DNA damage and cell proliferation induced by paracrine signals, and expansion of potential cells of origin for cancer. Future research using rodent prostatitis models should aim to distinguish the etiologies of BPH and PCa, and facilitate the development of novel clinical approaches for prostatic disease prevention.

## Introduction

Prostatitis is the inflammation of the prostate gland, and is characterized by immune cell (lymphocytes, neutrophils, macrophages, basophils, eosinophils) infiltration in the stromal compartment or localized regions surrounding the prostatic epithelial ducts. Often causing pelvic pain and sexual dysfunction, it is the most common urinary tract problem for men under the age of fifty (Collins et al., 1998). In the United States, prostatitis is estimated to account for two million visits to the clinics each year. Prostatitis is also gaining increasing attention because pathological and epidemiological evidence suggest that it is a significant etiologic factor in prostate cancer (PCa) (De Marzo et al., 2007; Sfanos et al., 2018). However, the mechanisms of prostatitis pathogenesis and its contribution to PCa development remain poorly understood. Vertebrate systems, particularly rodent models, provide invaluable tools to address these questions in the *in vivo* setting. By mimicking human prostatitis conditions and symptoms, those models allow for experimentation on various prostatic disease mechanisms and possible treatment options. In this review, we discuss commonly used vertebrate models of prostatitis in the field with a focus on their potential roles in elucidating the etiologic relationship of prostatitis, benign prostatic hyperplasia (BPH), and PCa.

### Rodent Models for Different Types of Prostatitis

Clinically, prostatitis can be divided into four types: acute bacterial inflammation, chronic bacterial inflammation, abacterial prostatitis or chronic pelvic pain syndrome (CPPS), and asymptomatic prostatic chronic inflammation (Vykhovanets et al., 2007; Gill and Shoskes, 2016; Liu et al., 2020). Prostatitis pathology differs among the types of inflammation and may be distinguished by immune cell types and their localization in different regions of the prostate (Sfanos et al., 2018). For example, acute inflammation usually features neutrophil infiltration, whereas chronic inflammation is mostly characterized by lymphocytes and macrophages (Sfanos et al., 2018; Ashok et al., 2019). Type IV or asymptomatic inflammation, due to its lack of symptoms in patients, can only be diagnosed based on increased leukocytes in biopsy samples taken after a prostate-specific antigen (PSA) test in prostate cancer screens (Porcaro et al., 2015). As a result, animal models of asymptomatic prostatitis are rare and difficult to define. In contrast, various methods, including bacterial infection, hormone treatment, immunization, stress, and diet manipulation, have been used to study acute and chronic bacterial prostatitis as well as CPPS in rodent models (Vykhovanets et al., 2007) (summarized in Table 1).

**TABLE 1:** Animal models of prostatitis and inflammation-associated BPH and PCa.

Model	References	Species, Strain	Histology
*Bacterial prostatitis by intraprostatic injection*	Olsson et al. (2012)	Rats, Sprague Dawley	Focal inflammation in dorsal-lateral prostate, diffuse and low inflammation in ventral prostate
	Xiong et al. (2017)	Rats, Sprague Dawley	Moderate to severe inflammation
*Bacterial prostatitis by intraurethral inoculation*	Boehm et al. (2012)	Mice, C57BL/6J	Significant acute inflammation, highest in anterior and dorsal-lateral prostate lobes
	(Elkahwaji et al., 2005, 2007, 2009)	Mice, BALB/c, C3H/HeJ, C3H/HeOuJ, C57BL/6J	Acute and chronic inflammation, hyperplasia, and PIN lesions
	Lilljebjörn et al. (2020)	Mice, C57BL/6	Mild acute and chronic inflammation
	Rippere-Lampe et al. (2001)	Rats, strain not specified	Moderate to high inflammation
	Shinohara et al. (2013)	Mice, C57BL/6J	Mild to chronic inflammation only in dorsal prostate
	Khalili et al. (2010)	Mice, C3H/HeOuJ	Acute inflammation and epithelial hyperplasia, ventral lobe most affected, lateral lobe least affected
	Kwon et al. (2013)	Mice, *K14-CreER; mTmG* and *K14-CreER; Pten* ^*fl/fl*^ *; mTmG*	Inflammation induced basal to luminal cell differentiation, accelerated tumor initiation
	Le Magnen et al. (2018)	Mice, *Nkx3.1* ^*−/−*^ and wildtype C57BL/6	Acute and chronic inflammation as well as hyperplasia, progression to PIN-lesions in *Nkx3.1* ^*−/−*^ mice
*Spontaneous CPPS*	Jackson et al. (2013)	NOD	Inflammation
	Penna et al. (2007a)	Mice, NOD	Chronic inflammation, more severe in aged mice
*Hormone-induced CPPS and BPH*	Konkol et al. (2019)	Rats, Wistar and Noble	Chronic inflammation, PIN-lesions and adenocarcinoma, Noble rats more susceptible than Wistar
	(J. Li J et al., 2018a)	Rats, Sprague-Dawley, and dogs, Beagle	Epithelial hyperplasia
	(Z. Li et al., 2018b)	Rats, Wistar	Epithelial hyperplasia
	Nicholson et al. (2012)	Mice, C57BL/6 and BALB/c	Increased prostate weight
	Yokota et al. (2004)	Dogs, Beagle	Epithelial hyperplasia
	Zou et al. (2017)	Mice, ICR	Epithelial hyperplasia
	(M. Zhang et al., 2020)	Rats, Sprague-Dawley	Hyperplasia, mild inflammation
	(Y. Li et al., 2019b)	Rats, Sprague-Dawley	Epithelial hyperplasia, mild inflammation
*Hormone + castration-induced CPPS*	Zang et al. (2021)	Rats, Sprague-Dawley	Chronic inflammation, testosterone increased and estradiol repressed prostate growth
	Jia et al. (2015)	Rats, Sprague-Dawley	Less inflammation but more hyperplasia with increasing testosterone doses
	Kamijo et al. (2001)	Rats, Wistar	Severe inflammation, stromal proliferation and fibrosis
	Tsunemori et al. (2011)	Rats, Wistar	Significant inflammation in ventral prostate lobe
*High-fat diet-induced CPPS and PCa*	Kwon et al. (2016)	Mice, C57BL/6	Inflammation and PIN-formation
	Shankar et al. (2012)	Mice, C57BL/6	Chronic inflammation
	(H. Xu et al., 2015)	Mice, TRAMP	High-fat diet increased mortality and tumor formation rate in the TRAMP model
*EAP by LPS or autoantigen injection*	dos Santos Gomes et al. (2017)	Mice, Swiss and C57Bl/6	Inflammation, hyperplasia
	(D. Xu et al., 2019)	Rats, Sprague-Dawley	Inflammation, hyperplasia
	Kim et al. (2013)	Rats, Sprague-Dawley	Inflammation, hyperplasia
	Jackson et al. (2013)	Mice, Balb/c, B10.D2, NOD, SWR, MRL and NZB	Chronic inflammation in Balb/c, minor in SWR, acute inflammation resolved in NZB
	Penna et al. (2007b)	Mice, NOD	Chronic inflammation, slightly more severe immune cell infiltration when injected with MAG instead of just PSBP
	Popovics et al. (2017)	Mice, BALB/c	Chronic inflammation and hyperplasia
	(X. J. Wang et al., 2016)	Rats, Sprague-Dawley	Chronic inflammation and hyperplasia
	(M. Zhang et al., 2020)	Rats, Sprague-Dawley	Severe inflammation, moderate hyperplasia
*POET model for CPPS*	Burcham et al. (2014)	Mice, POET-3	Chronic inflammation and rare hyperplastic lesions
	Haverkamp et al. (2011)	Mice, POET-3	Severe acute inflammation
	Lees et al. (2006)	Mice, POET-1 and POET-3	Mild to moderate acute inflammation
	(H. H. Wang et al., 2015)	Mice, POET-3	No histological images
*CPPS in other GEMMs*	Ashok et al. (2019)	*Hoxb13-rtTA; TetO-IL1B*	IL1b overexpression, acute and chronic inflammation, epithelial proliferation, fibrosis
		Mice, FVB/N	
	Liu et al. (2017)	*Pb-IL6* transgenic mice, C57BL/6	IL6 overexpression, infiltrating inflammatory cells, PIN-lesions and adenocarcinoma
	Pascal et al. (2021a)	*PSA-CreER; Cdh1* ^*fl/fl*^ mice, C57BL/6J	Deletion of E-cadherin, inflammation, hyperplasia and fibrosis in all lobes
	(B. Zhang et al., 2016)	*K8-CreER; AR* ^*fl/Y*^ mice	Luminal AR deletion, up-regulation of inflammatory cytokines and down-regulation of tight-junction proteins
	Hou et al. (2009)	*Aire-KO* Mice, B6 and NOD Lt/J backgrounds	Moderate to severe inflammation

Bacterial infection is frequently used to study acute and chronic bacterial inflammation and is induced either by direct injection of uropathogenic bacteria into the prostate lobes of rodents (Olsson et al., 2012; Xiong et al., 2017) or by inoculation via an intraurethral catheter (Rippere-Lampe et al., 2001; Elkahwaji et al., 2005; Elkahwaji et al., 2007; Elkahwaji et al., 2009; Khalili et al., 2010; Boehm et al., 2012; Shinohara et al., 2013; Le Magnen et al., 2018; Lilljebjörn et al., 2020). Different rodent species and strains have been used, including Wistar and Sprague-Dawley rats and C57BL/6 and C3H/HeJ mice. While some of the infected rodents recover spontaneously, many will develop chronic inflammation following initial acute inflammation response (Vykhovanets et al., 2007). Commonly used bacterial strains for infection include various uropathogenic *Escherichia coli* strains (Rippere-Lampe et al., 2001; Elkahwaji et al., 2005; Elkahwaji et al., 2007; Elkahwaji et al., 2009; Boehm et al., 2012; Lilljebjörn et al., 2020), as well as other species such as *Propionibacterium acnes* (Olsson et al., 2012; Shinohara et al., 2013). Although *P. acnes* infection might take longer to induce inflammation compared to *E. coli,* both can induce acute and chronic inflammation, with lesions featured by higher cell proliferation and diminished Nkx3.1 and androgen receptor (AR) expression (Shinohara et al., 2013). Clinically, chronic bacterial prostatitis is often developed from acute bacterial prostate inflammation. Therefore, these infection models are highly relevant as they mimic disease etiology.

In contrast to bacterial inflammation, the direct cause of abacterial prostatitis/CPPS remains unclear (Liu et al., 2020; Tsunemori and Sugimoto, 2021). Possible disease mechanisms include physical and chemical damage by urine reflux, sexually transmitted pathogens, diet, hormone imbalances, and autoimmunity (De Nunzio et al., 2011). Consequently, a wide range of animal models has been developed to explore the many potential causes of CPPS. Notably, certain rodents such as Wister, Lewis and Copenhagen rats, develop abacterial chronic prostatitis spontaneously as they age (Lundgren et al., 1984; Sharma et al., 1992; Keith et al., 2001). In men, aging is associated with increased prevalence of CPPS and a decline of the serum testosterone to estradiol ratio (T-to-E2 ratio) (Harman et al., 2001; Bernoulli et al., 2008). One proposed mechanism is that a decreased T-to-E2 ratio disrupts the balance between the immunosuppressive effect of testosterone and the pro-inflammatory effect mediated by estrogen (Cutolo et al., 2002). To mimic this, hormone-induced animal models of CPPS are often based on decreasing the T-to-E2 ratio, either by administration of estradiol or a combination of estradiol and testosterone (Kamijo et al., 2001; Tsunemori et al., 2011; Jia et al., 2015; Konkol et al., 2019; Zang et al., 2021). In other approaches, a high fat diet (HFD) has been shown to induce chronic inflammation in rodents (Shankar et al., 2012; Shankar et al., 2015; Xu et al., 2015; Kwon et al., 2016). HFD-induced oxidative stress and NF-κB and Stat3 signaling activation may play important roles in this process (Shankar et al., 2012; Shankar et al., 2015), but the mechanisms by which HFD promotes chronic inflammation remain to be fully elucidated.

One of the great advantages of using mouse models is the capability of genetically manipulating gene expression *in vivo*. Several genetically engineered mouse models (GEMMs) have been reported to be able to induce chronic inflammation. These include prostate-specific knockout of the gene encoding AR or E-cadherin (Zhang et al., 2016; Pascal et al., 2021b), which increases prostate epithelial barrier permeability. Genetically modified mice are particularly useful for modeling immune-related chronic prostate inflammation, whose phenotypes are commonly referred to as experimental autoimmune prostatitis (EAP). For example, an inherent lack of immunity can cause chronic prostatitis in aging NOD mice, a strain prone to developing organ-specific autoimmune disease (Kikutani and Makino, 1992). In these models, the autoimmune origin is evident by a T-cell response to prostate autoantigens and characterized by CD4^+^ T-cell intraprostatic infiltration (Penna et al., 2007a; Jackson et al., 2013). Other genetic models include overexpression of the pro-inflammatory cytokines IL-1β or IL-6 in transgenic mice (Liu et al., 2017; Ashok et al., 2019), and the *Aire*-deficient mouse model, in which knockout of the important immune regulator *Aire* led to development of chronic prostatitis (Hou et al., 2009).

Notably, EAP can also be triggered by injection of lipopolysaccharide (LPS), a component of the Gram-negative bacterial cell wall, which stimulates the release of pro-inflammatory cytokines to induce chronic inflammation (Kim et al., 2013; dos Santos Gomes et al., 2017; Xu et al., 2019). Other EAP models induce inflammation by injecting a combination of autoantigens with an adjuvant. These autoantigens are prostate specific, such as male accessory gland extract (Jackson et al., 2013) and prostate tissue homogenate (Wang et al., 2016; Popovics et al., 2017). However, as homogenized tissue contains multiple antigens, this makes some of these immunological models unfit to study T-cell/antigen specific interactions. Furthermore, many models use endogenous T-cell pools that have had previous antigen exposure, further limiting specificity of the T-cell response (Lees et al., 2006). Consequently, the prostate ovalbumin-expressing transgenic (POET) mouse model was developed as an antigen-specific autoimmune model of both acute and chronic prostate inflammation (Lees et al., 2006; Haverkamp et al., 2011; Burcham et al., 2014; Wang et al., 2015). Using the ARR_2_PB promoter, POET mice express high levels of membrane-bound ovalbumin in the different lobes of the prostate (Lees et al., 2006; Haverkamp et al., 2011). Using adoptive transfer of transgenic T-cells that recognize ovalbumin, the POET model circumvents general tolerance mechanisms and provides the opportunity to monitor a specific T-cell population during both chronic and acute prostate inflammation.

### Vertebrate Models That Involve Inflammation and Benign Prostatic Hyperplasia

Benign prostatic hyperplasia (BPH) is a condition in which hyperplasia of the stromal and glandular prostatic cells causes prostate enlargement (Nickel, 2008). Clinically, BPH is characterized by lower urinary tract symptoms (LUTS) such as voiding, storage and post-micturition symptoms, and can be associated with bladder outlet obstruction (BOO) (Roehrborn, 2005; Chughtai et al., 2011). Some BOO animal models involve mechanical obstruction of the urethra by sutures or ligatures, thus directly affecting urine outflow (Austin et al., 2004; Kanno et al., 2016). However, the relevance of these models to BPH-induced BOO is unclear due to the invasiveness of the procedure and the fact that BPH is a disease that develops over a long period of time. Rather, animal models that recapitulate age-related spontaneous BPH development are desired. In contrast to spontaneous prostatitis models, only macaques, chimpanzees, and dogs are known to naturally develop BPH, with dogs being the most commonly used animal model for BPH (Sun et al., 2017; Zhang et al., 2021). Interestingly, since age-related change in hormone ratios is thought to contribute to BPH development, rodent models of BPH have been developed by castration and administration of testosterone and/or estrogen (Yokota et al., 2004; Nicholson et al., 2012; Zou et al., 2017; Li J. et al., 2018; Li Z. et al., 2018; Li Y. et al., 2019; Zhang et al., 2020), similar to the hormone-induced CPPS rodent models (Kamijo et al., 2001; Tsunemori et al., 2011; Jia et al., 2015; Konkol et al., 2019). Indeed, many aforementioned chronic prostatitis models also show BPH phenotypes. For example, high-fat diet as well as LPS and *E. coli* injection can induce both inflammation and BPH phenotypes in rodents (Elkahwaji et al., 2007; Escobar et al., 2009; Shankar et al., 2012; Kim et al., 2013; Kwon et al., 2016; dos Santos Gomes et al., 2017; Li Y. et al., 2019; Xu et al., 2019), and the EAP model used to induce chronic prostatitis can induce BPH in rats (Wang et al., 2016; Zhang et al., 2020). This overlap between animal models for prostatitis and BPH is reflected in clinical findings: biopsies taken from patients with BPH often show immune cell infiltration and markers of inflammation (Taoka et al., 2004; Penna et al., 2007b; Nickel, 2008; Robert et al., 2009; Taoka and Kakehi, 2017). Similarly, several studies found bacterial and viral strains in BPH specimens, suggesting that bacterial inflammation may play a role in BPH development (Nickel et al., 1999; Chughtai et al., 2011).

Mechanistically, it has been postulated that chronic inflammation can create a microenvironment that induces wound healing repair processes, leading to the activation of proliferative pathways and hence prostate hyperplasia (Taoka et al., 2004; Fibbi et al., 2010). For example, inflammation-induced leakage of the epithelial barrier could lead to an influx of luminal-secreted autoantigens into the stromal compartment and subsequently produce an autoimmune response (Chughtai et al., 2011; Li F. et al., 2019; Pascal et al., 2021b). Indeed, PSA has been detected in stroma surrounding BPH nodules from patients (O'Malley et al., 2014), and a large scale analysis of BPH patient tissues revealed that high serum PSA values were associated with inflammation (Gandaglia et al., 2013). One possible mechanism of epithelial barrier leakage may be through down-regulation of E-cadherin, an important regulator of the epithelial barrier and tissue homeostasis. E-cadherin expression is often found to be lower in BPH tissues (Kim et al., 2013; Li F. et al., 2019; Xu et al., 2019; Pascal et al., 2021a), and conditional knockout of E-cadherin in the mouse prostate causes loss of epithelial barrier function, inflammation and hyperplasia (Pascal et al., 2021b). Additionally, conditional overexpression of the pro-inflammatory cytokine interleukin-6 (IL-6) down-regulates E-cadherin (Liu et al., 2017), suggesting that a positive feedback loop between inflammation and epithelial barrier disruption may be present to promote BPH. Similarly, a recent study showed that attenuation of luminal epithelial AR signaling can induce prostate inflammation and impair epithelial cell tight junctions, while inflammation can suppress AR expression (Zhang et al., 2016). Such a positive feedback loop may also be involved in sustaining chronic inflammation during BPH progression. Despite these progresses, whether inflammation directly causes BPH or is an associated factor during BPH progression remains unclear. Further research is needed to clarify the relationship between chronic prostatitis and BPH.

### Rodent Models for Studying the Relationship Between Prostatitis and Prostate Cancer

**FIGURE 1:**
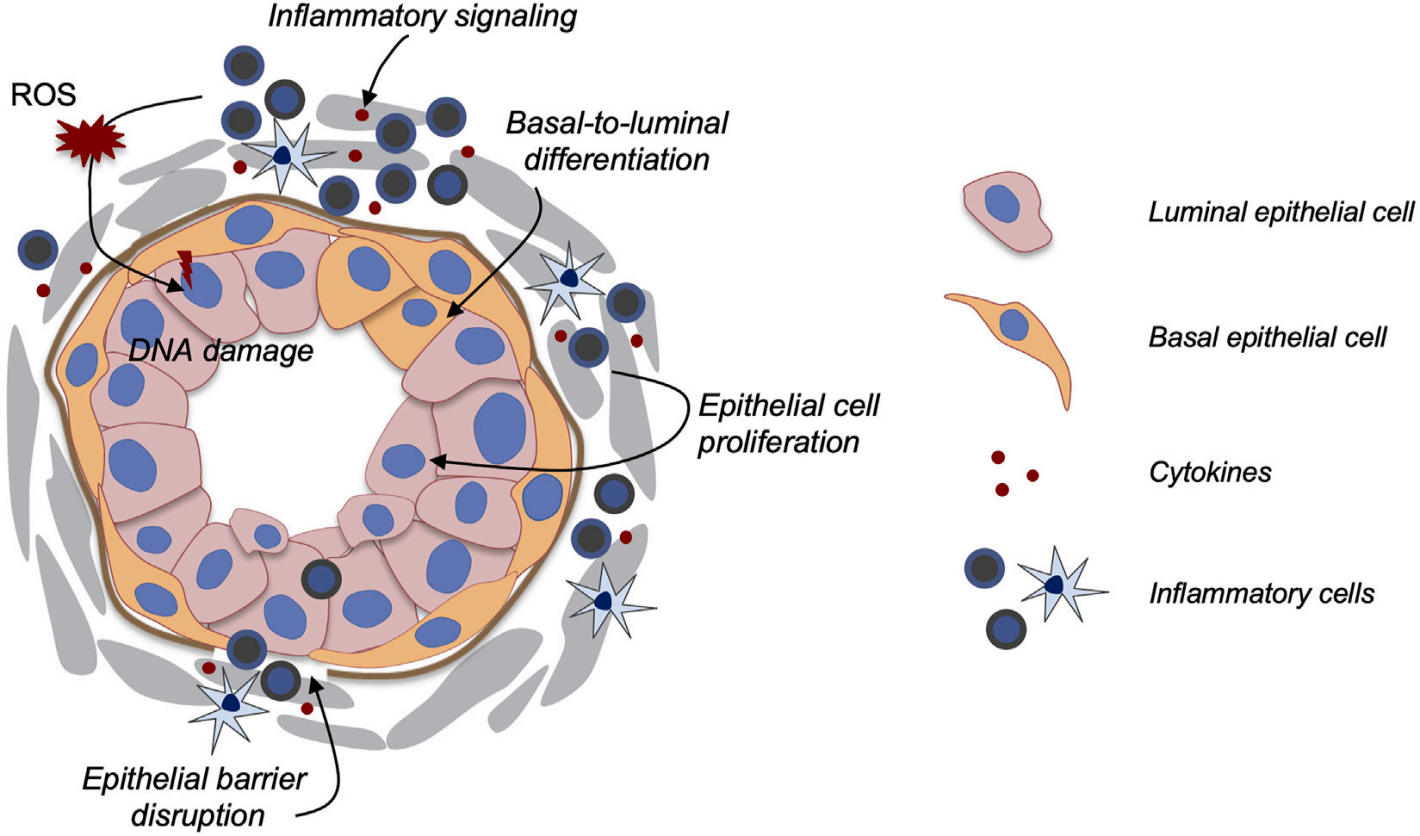
Model of how prostatitis promotes PCa. Multiple possible mechanisms have been proposed as illustrated in the diagram. Secreted cytokines may promote epithelial cell proliferation, basal-to-luminal cell differentiation, and epithelial barrier disruption. Inflammation-induced ROS production and *Nkx3.1* down-regulation could also enhance DNA damage.

Prostate cancer (PCa) is the second leading cause of cancer-related morbidity and mortality in American men. The etiologic link between prostatitis and PCa has long been suggested (De Marzo et al., 2007; Sfanos et al., 2018). For example, in human prostatectomy specimens, lesions characterized by proliferating epithelial cells and activated inflammatory cells (named proliferative inflammatory atrophy, PIA) are often adjacent to areas of prostatic intraepithelial neoplasia (PIN) (De Marzo et al., 1999). Recently, inflammation in benign tissues identified in the Prostate *Cancer* Prevention Trial was positively associated with later development of PCa (Platz et al., 2017), strongly suggesting that chronic prostatitis is a precursor of PIN and PCa. To date, however, the mechanisms linking prostatitis and PCa development remain unclear. Uncovering these mechanisms should aid PCa prevention and early intervention. Below, we discuss three major possible avenues of how prostatitis may facilitate PCa progression (Figure 1) with a focus on applications of mouse models: 1) enhanced secretion of cytokines and growth factors to promote epithelial cell proliferation, 2) inflammation-induced epithelial cell DNA mutations, and 3) increasing basal-to-luminal differentiation to enlarge the pool of cells of origin for PCa.

#### Enhanced Secretion of Cytokines and Growth Factors to Activate Epithelial Cell Proliferation

The mechanisms by which inflammation contributes to PCa development are multifaceted. One of the more direct ways may be through activating epithelial cell proliferation via paracrine signals from the inflammatory stroma. The normal prostate mostly contains quiescent cells, while cell proliferation is necessary for tissue wound healing. Interestingly, the reactive stroma observed in BPH and PCa undergoes changes resembling a wound healing response (Tuxhorn et al., 2001; Schauer and Rowley, 2011). Infiltration of inflammatory cells, increased growth factor availability, angiogenesis, and extracellular matrix remodeling are among the major features of such a pro-tumor microenvironment. The infiltrating inflammatory cells can produce a wide range of cytokines such as tumor necrosis factor (TNF) and interleukins (ILs), which can induce further secretion of growth factors to promote epithelial cell proliferation (Giri and Ittmann, 2001; Steiner et al., 2002; Sokol and Luster, 2015). For example, an *in vitro* study showed that in prostate epithelial cells, cytokines secreted by macrophages could activate ERK and Akt, two protein kinases that promote cell proliferation and survival (Dang and Liou, 2018). Furthermore, GEMMs offer great models to study the effects of inflammatory signaling on the prostate *in vivo*. In particular, overexpression of human IL-6 in the mouse prostate showed development of chronic inflammation and progressive neoplasia, with PIN lesions and prostate adenocarcinoma observed later (Liu et al., 2017). Moreover, in the genetic mouse prostatitis model where interleukin 1β (IL-1β) is overexpressed, increased expression of downstream cytokines were observed, along with formation of PIA-like lesions and high expression of the proliferation marker Ki67 (Ashok et al., 2019). As discussed previously regarding inflammation and BPH, these genetic mouse models suggest the involvement of a positive feedback loop between inflammation and epithelial barrier disruption to promote cell proliferation, as evidenced by the down-regulation of E-cadherin in the IL-6 overexpression model (Liu et al., 2017). However, it is important to note that cancer development requires more than just cell proliferation. Additional inflammation-induced mechanisms must be in play to explain the phenotypic differences between BPH and PCa.

#### Inflammation-Induced Oxidative Stress and Loss of Nkx3.1 can Induce DNA Damage

Studies across many organ types have suggested that inflammation can increase genomic instability (Colotta et al., 2009; Grivennikov et al., 2010). One proposed mechanism is the release of reactive oxygen species (ROS) by infiltrating inflammatory cells. ROS, such as superoxide, nitric oxide and hydrogen peroxide, are highly reactive oxygen-containing molecules that are produced during natural metabolic processes (Ihsan et al., 2018). Excess ROS production can result in an imbalance between ROS and antioxidants, leading to insufficient ROS degradation. This state of oxidative stress can cause oxidative damage in DNA, RNA, proteins, and lipids (Olinski et al., 2002; Lugrin et al., 2014; Ihsan et al., 2018). Notably, although much focus is placed on DNA damage, proteins and lipids are also important targets for oxidative attack, as modification of these molecules can increase the risk of mutagenesis (Reuter et al., 2010; Murata, 2018). Continuous exposure to inflammation and concurrent immune cell infiltration can lead to increased levels of ROS (Xia and Zweier, 1997; Eiserich et al., 1998), which can lead to genetic mutations and instability (Weitzman and Stossel, 1981; Weitzman and Gordon, 1990). It is hypothesized that in PIA lesions, where inflammatory injury stimulates epithelial cell proliferation, ROS released by infiltrating inflammatory cells can increase formation of PIN-lesions and carcinoma (Wiseman and Halliwell, 1996; Xia and Zweier, 1997; De Marzo et al., 1999). Using animal models, a mechanistic link between oxidative stress and inflammation has been established in mice susceptible to colon inflammation, in which knockout of *Gpx1* and *Gpx2*, two genes that encode antioxidant enzymes, results in a high incidence of tumors in the intestinal epithelium (Chu et al., 2004). However, similar models in the PCa context are currently lacking. To functionally test the role of ROS in promoting inflammation-induced PCa, it will be very informative to genetically perturb the ROS production pathway in mice or combine ROS production perturbation with other oncogenic pathways to assess the effect on PCa development.

Genetic mouse model studies also suggested that another possible mechanism of inflammation-induced epithelial DNA damage could be related to *Nkx3.1* down-regulation. Nkx3.1, besides serving as a transcription factor in prostate development, is also a tumor suppressor (Bhatia-Gaur et al., 1999; Kim et al., 2002). Its tumor suppressing functions can at least be partially attributed to its role in preventing DNA damage (Bowen and Gelmann, 2010; Bowen et al., 2013; Debelec-Butuner et al., 2015). PIN formation in *Nkx3.1* ^*−/−*^ mice was reported to be associated with deregulation of prooxidant and antioxidant enzymes, as well as oxidative damage in DNA (Ouyang et al., 2005). Notably, acute bacterial prostatitis in mice leads to down-regulation of Nkx3.1 (Khalili et al., 2010; Shinohara et al., 2013), and lower Nkx3.1 expression was also observed in the genetic prostatitis model of IL-1 overexpression (Ashok et al., 2019). Moreover, inducing prostate inflammation in *Nkx3.1* ^*−/−*^ mice accelerates PCa initiation (Le Magnen et al., 2018). These findings suggest that there may be a positive feedback or inflammatory storm mechanism at play. In such a model, inflammatory cytokines such as TNF-α and IL-1β could stimulate Nkx3.1 down-regulation (Markowski et al., 2008; Debelec-Butuner et al., 2014), which in turn would increase susceptibility to oxidative stress and further DNA damage (Ouyang et al., 2005). Such a combined environment of inflammatory signaling, oxidative stress, and high epithelial proliferation, could give rise to PIN and PCa (De Marzo et al., 1999).

#### Inflammation-Induced Basal-To-Luminal Differentiation Expands Cells of Origin for PCa

Cell of origin for PCa has been implicated as a link between prostatitis and PCa. A cell of origin is defined as a normal tissue cell that can give rise to a tumor after its oncogenic transformation (Blanpain, 2013; Lee and Shen, 2015). Tissue stem cells, due to their self-renewal and multipotent capabilities, can serve as potent cells of origin for cancer. In an earlier colon cancer study, inflammation induces tissue stem cell expansion, potentially enlarging the cellular pool for oncogenic transformation (Umar et al., 2009). In the prostate, lineage-tracing studies in mice have shown that epithelial basal cells are the stem cells that can generate luminal cells during prostate organogenesis (Ousset et al., 2012). However, basal stem cell activities become restricted in the mature prostate as basal and luminal cells are mostly two self-sustained lineages at adulthood and basal-to-luminal cell differentiation is rare (Choi et al., 2012; Wang et al., 2013). Importantly, basal-to-luminal differentiation appears to be an important step towards PCa initiation. In mouse lineage-tracing models, loss of the tumor suppressor gene *Pten* in basal cells promoted basal-to-luminal differentiation, and the resulting tumor had a luminal phenotype (Choi et al., 2012; Wang et al., 2013), resembling the predominant luminal feature in human PCa (Shen and Abate-Shen, 2010). In fact, loss of the basal cell layer is often considered a hallmark of PCa (Humphrey, 2007; Grisanzio and Signoretti, 2008). We previously showed that luminal cells are the favored cell type of origin for PCa (Wang et al., 2014). Therefore, by enhancing basal cell plasticity and basal-to-luminal differentiation, the cellular pool for oncogenic transformation is enlarged, potentially facilitating PCa development.

In light of this, it is particularly interesting to note that basal-to-luminal differentiation was reported to be enhanced in two prostatitis mouse models. When mice were either inoculated with uropathogenic *E. coli* (UPEC) or fed with HFD, basal cells rapidly proliferated and produced luminal cells (Kwon et al., 2013; Kwon et al., 2016). Both treatments also accelerated PCa initiation in the basal-specific *Pten*-knockout model (*K14-Pten*) (Kwon et al., 2013; Kwon et al., 2016). PCa developed relatively slowly in basal-specific *Pten*-knockout models since it takes time for *Pten* deletion to drive basal cells towards transformed luminal cells (Choi et al., 2012; Wang et al., 2013). *K14-Pten* mice treated with UPEC or HFD showed accelerated disease progression, indicating that faster basal-to-luminal differentiation due to inflammation-induced signals facilitated PCa development. In the future, identifying those signals that promotes basal-to-luminal differentiation should be beneficial for delaying PCa progression in patients with chronic prostatitis.

## Conclusion and Discussion

Numerous rodent models have been developed to study the different types of clinically defined prostatitis. While bacterial prostatitis models have recapitulated many aspects of the acute and chronic bacterial inflammation observed in humans, it remains challenging to pinpoint the most relevant model for CPPS, since the molecular pathways responsible for abacterial chronic prostatitis are not yet fully understood. The etiology of chronic prostate inflammation can vary among individuals, and different CPPS models, including hormone, high fat, autoimmune, and GEMMs may capture different important aspects of CPPS development. These animal models have been playing crucial roles in our efforts to elucidate the relationship between prostatitis and other prostatic diseases such as BPH and PCa. The association of prostatitis to these diseases is well documented in clinical studies. In recent years, applications of GEMM prostatitis models have revealed possible mechanisms by which inflammation causes BPH and PCa. Among those mechanisms, disruption of the epithelial barrier and the ensuing auto feedback loop of enhanced inflammation appear to be a common theme. Nonetheless, inflammation may contribute to PCa development in many other ways, such as oxidative stress-induced DNA damage, down-regulation of the tumor suppressor Nkx3.1, and expansion of luminal epithelial cells as cells of origin. Future research utilizing rodent models will continue to shed light on the mechanistic, causal links between chronic prostate inflammation and progressive prostatic diseases, and should distinguish the etiology between BPH and PCa. Such insights will be invaluable for prostatic disease prevention and early intervention.
